# Interleukin-2 Functions in Anaplastic Large Cell Lymphoma Cells through Augmentation of Extracellular Signal-Regulated Kinases 1/2 Activation

**Published:** 2011-09

**Authors:** Masanori Ito, Nianxi Zhao, Zihua Zeng, Xiaobo Zhou, Chung-Che Chang, Youli Zu

**Affiliations:** *Department of Pathology, the Methodist Hospital and the Methodist Hospital Research Institute, Houston, TX, USA*

**Keywords:** anaplastic large cell lymphoma (ALCL), CD25/IL-2 receptor, IL-2 signaling, extracellular signal-regulated kinases (ERK1/2), tumor microenvironment

## Abstract

In addition to intrinsic genetic alterations, the effects of the extrinsic microenvironment also play a pathological role in cancer development. Altered chemokine/cytokine networks in the tumor microenvironment may contribute to the dysregulation of cellular functions in cancer cells. Anaplastic large cell lymphoma (ALCL) is an aggressive T-cell lymphoma caused by abnormal expression of anaplastic lymphoma kinase due to a chromosomal translocation. Notably, ALCL cells are also characterized by high-level expression of the high-affinity IL-2 receptor subunit CD25 on the cell surface. However, whether the IL-2/IL-2 receptor functions in ALCL cells and how this signaling affects the tumor remain unclear. In this study, we treated cultured ALCL cells with exogenous IL-2 and examined changes in cellular function and signaling pathways. IL-2 stimulated cell growth and augmented activation of the extracellular signal-regulated kinases 1/2 (ERK1/2) pathway. Additionally, IL-2 enhanced lymphoma cell survival by overcoming kinase inhibitor U0126-induced growth arrest and apoptosis. Subsequently, to identify the potential source of IL-2 for lymphoma cells *in vivo*, we performed gene expression and immunochemical analyses. RT-PCR revealed no IL-2 gene expression in cultured ALCL cells and ruled out the possibility of an IL-2 autocrine loop. Interestingly, immunostaining of lymphoma tumor tissues showed IL-2 protein expression in background cells within tumor tissue, but not in ALCL cells. Our findings demonstrate that IL-2 signaling plays a functional role in ALCL cells, and enhances lymphoma cell survival by increasing activation of the ERK1/2 pathway.

## INTRODUCTION

Lymphomas are cancers that originate from cells of the lymphatic system. Similar to other cancers, lymphoma development results from a combination of intrinsic events and extrinsic microenvironment pathogenic effects, including cytokine- and/or stromal cell-induced effects on lymphoma cells (1, 2). In addition, survival and proliferation of lymphoma cells are also dependent on the interactions with their microenvironment (3-5). The presence of an altered chemokine/cytokine network within the tumor microenvironment may further regulate lymphoma cell functions including proliferation, apoptosis, migration, and angiogenesis (6, 7). Hodgkin lymphoma (HL) is an example of how tumor cells interact with the microenvironment for their survival (8). It has been reported that HL tumor cells can express and secrete a variety of cytokines, chemokines, and chemokine ligands, and that the background cells within the tumor microenvironment also secrete cytokines (9-12). Importantly, the tumor microenvironment provides growth factors and stimuli via receptor-ligand interactions that regulate HL cell biology and support survival of HL cells by protecting them from apoptosis (8). Moreover, increased expression of the chemokines CXCL12 and CXCL13 in the tumor microenvironment has been shown to enhance the migration of follicular lymphoma cells (13, 14). Anaplastic lymphoma kinase (ALK)-positive anaplastic large T-cell lymphoma (ALCL) is characterized by anaplastic cytology and pathogenic expression of the ALK gene due to a chromosomal translocation (15-18). ALCL cells express high levels of CD30, a member of the tumor necrosis factor receptor superfamily that activates cellular NF-κB pathways (19) and regulates JunB expression (20, 21). Interestingly, ALCL cells also express high levels of CD25 (18), a subunit of high-affinity Interleukin-2 receptors (22, 23). However, the potential pathophysiological role of CD25 expression in ALCL is not yet known.

Interleukin-2 (IL-2), a growth factor derived from activated T lymphocytes, functions via its receptor on the cell surface (24, 25). Three distinct classes of IL-2 receptors exist: those that bind IL-2 with low, intermediate, and high affinity (26). The high-affinity receptors mediate the majority of IL-2-dependent effects, and the function of these receptors is controlled by expression of the CD25 gene, a key subunit of the high-affinity receptors (22, 23). The interaction of IL-2 with its high-affinity receptor is a critical control point in the T cell and natural killer (NK) cell-mediated immune response and their proliferation under normal physiologic conditions (27). Molecular studies have revealed that IL-2-induced cellular functions are mediated via multiple intracellular signaling pathways that include extracellular signal-regulated kinases 1/2 (ERK1/2), Akt kinase, signal transducer and activator of transcription STAT3, and STAT5 (28-32). Notably, IL-2 and/or IL-2 receptor are also involved in certain pathogenic processes. *In vitro*, activation of the IL-2 and IL-2 receptor promoters is mediated by cellular transfection of human T-cell leukemia virus type I (HTLV-I), a pathogenic factor in human adult T-cell leukemia/lymphoma (ATLL) (33-37). Studies of leukemic cells from ATLL patients demonstrated abnormally high levels of expression of IL-2 receptor (38-40). In addition, infection of an IL-2-dependent murine cytotoxic T-cell line with a retrovirus vector carrying the human IL-2 gene resulted in factor-independent growth *in vitro* and tumorigenicity *in vivo* (41). The generation of cells that constitutively express IL-2 receptor and secrete IL-2 revealed that the transfected cells proliferated *in vitro* autonomously through an IL-2 autocrine mechanism and that the higher producers of IL-2 developed tumors *in vivo* (42). Taken together, these accumulated data indicate that dysregulation of the IL-2 pathway and/or the aberrant activation of the IL-2 autocrine loop are involved in the development of ATLL by the infection of CD4+ helper T cells with HTLV-I (43-47). However, whether IL-2 signaling has functional consequences in ALCL cells has not yet been explored, despite the high-level expression of surface CD25 in tumor cells. In this study, we investigated functions of IL-2 signaling in cultured ALCL cells and examined potential sources of IL-2 within lymphoma tumors.

## MATERIALS AND METHODS

### Cell lines, reagents, and cell function assays

Human ALCL cell lines (Karpas 299 and SUDHL-1) were obtained from the German Collection of Microorganisms and Cell Cultures (DSMZ). Both cell lines carry the chromosomal translocation t (2; 5) (p23; q35) and express the oncogenic nucleophosmin (NPM)/ALK fusion protein (48-50). Jurkat (Leukemia) and RPMI 8226 (Myeloma) from the American Type Culture Collection (ATCC) were used as CD25 negative control. All cell lines were routinely maintained in RPMI 1640 medium supplemented with 5% fetal calf serum (FCS). Recombinant human IL-2 was purchased from Chemicon International Inc. (Temecula, CA). To inhibit the ERK1/2 pathway, U0126 (Tocris Bioscience, Ellisville, MI), a kinase inhibitor specific for the upstream mitogen activated kinase kinase (MEK1/2), was used (51). For cell stimulation, Phorbol 12-myristate 13-acetate (PMA) and concanavalin A (ConA) were obtained from Sigma (St. Louis, MO).

To study the potential cellular effects of IL-2, ALCL cells (1 × 10^5^ cells/ml) were cultured in RPMI 1640 with 5% or 0.5% FCS to minimize potential serum-derived factor effects on cell growth and proliferation. The cells were treated with increasing amounts of recombinant human IL-2 (from 1 to 40 units/ml) as indicated in the figures. After 5 days, cells were harvested and stained in phosphate-buffered saline (PBS) containing 0.1% trypan blue. The number of viable cells in each tested condition was determined using a hemocytometer with a light microscope. The relative number of viable cells was determined by calculating the ratio of viable cells in cultures containing IL-2 to control cells in cultures without IL-2. To downregulate the ERK1/2 pathway, cells (2.5 × 10^5^ cells/ml) cultured in RPMI 1640 containing 5% FCS were exposed to the kinase inhibitor U0126 (5 μM) in the presence or absence of IL-2 (40 units/ml) for 36 hours, and viable cells were counted as described above. For apoptosis assays, treated cells were fixed and stained with FITC-conjugated annexin V and propidium iodide according to the manufacturer’s instructions (BD Biosciences, San Diego, CA). Apoptotic cells (%) were detected by flow cytometric analysis.

### Western blotting of cellular protein kinases

Antibodies for human ERK1/2, phosphorylated ERK1/2, and NPM-ALK proteins were purchased from Cell Signaling (Beverly, MA). Western blotting was performed on extracts from Karpas 299 and SUDHL-1 cells (5 × 10^6^ cells/sample) treated with IL-2 (40 units/ml) and/or exposed to U0126 (5 μM) for 30 minutes. The cells were harvested, and cellular proteins were separated by 10% SDS-PAGE and transferred to nitrocellulose membranes. Blots were probed with the primary antibodies followed by a peroxidase-conjugated goat anti-rabbit secondary antibody (Sigma, St. Louis, MO). The proteins of interest were detected using luminol chemiluminescent detection reagents (Sigma).

### Flow cytometric analysis for cell-surface CD25 expression

Karpas 299 and SUDHL-1 cells were stained using PerCP-Cy5 fluorescent-conjugated antibodies specific for human CD25 proteins (BD Biosciences, San Diego, CA) and analyzed by flow cytometry. As a negative control, Jurkat cells, a human T-cell leukemia line that expresses little to no CD25, were also stained.

### Immunohistochemical staining

Formalin-fixed and paraffin-embedded tissue sections of human systemic ALCL tumors from two patients were retrieved from archival files in the Department of Pathology and Laboratory Medicine of The Methodist Hospital and stained with hematoxylin and eosin (H & E). Expression of CD25 and CD30 in lymphoma cells, and CD3 in background T cells, was confirmed by immunohistochemical (IHC) studies with a mouse anti-human antibodies (clone 4C9 for CD25; Vector Laboratories, Burlingame, CA; antibodies for CD3 and CD30 from Dako, Carpinteria, CA) following standard laboratory protocols. To detect protein expression of IL-2, goat anti-human/rat IL-2 antibody was used (Neuromics, Edina, MN; Cat#: GT15155) in a modified IHC procedure. Briefly, deparaffinized tissue sections were incubated in citrate buffer (pH6.0) in a water bath at 95°C for 10 minutes for antigen retrieval, according to the manufacturer’s instructions. The sections were then washed 3 times with PBS buffer (pH7.4) for 3 minutes each, and the endogenous peroxidase activity in the specimens was blocked with 3% H_2_O_2_ for 20 minutes at room temperature. The IL-2 antibodies (1:400 dilution) were added to the tissue sections and incubated in a humidified chamber at room temperature overnight. After 3 washes with PBS, the specimens were incubated with the secondary antibodies (horseradish peroxidase [HRP]-conjugated rabbit anti-goat IgG) at room temperature for 45 minutes. Finally, proteins were visualized by adding 3.3’-diaminobenzidine (DAB), the substrate for HRP, for approximately 5 minutes. Counterstain of stained tissue was then performed with hematoxylin for 5 seconds. The positive immunoreaction for CD3, CD25, and CD30 was visualized by the addition of peroxidase-conjugated horse anti-mouse Ig antibodies and color development reagents according to the manufacturer’s instructions (Vector Laboratories, Burlingame, CA).

To examine IL-2 protein expression in cultured ALCL cell lines, the cultured Karpas 299 and SUDHL-1 cells were treated with 10 nM of PMA overnight. Cells were re-suspended in 0.15 ml plasma and mixed with 0.15 ml of thrombin solution at room temperature for 5 minutes. The formed cell blocks were then fixed in formalin and embedded in paraffin following routine histology procedures. Immunostaining for IL-2 protein was then performed on sections of the cell blocks as described above.

### RT-PCR study of cellular IL-2 gene expression

Karpas 299 and SUDHL-1 cells were stimulated with 10 nM of PMA, 50 ng/ml of ConA, or vehicle control for 3 hours. The cells (1 × 10^7^ cells/sample) were harvested, and total cellular RNA was isolated using the Trizol reagent according to the manufacturer’s instructions (Invitrogen, Carlsbad, CA). The purified RNAs were denatured at 70°C for 10 min and reverse-transcribed with random hexamers at 42°C for 1 hour using a Superscript III kit (Invitrogen, Carlsbad, CA). The IL-2 gene was amplified using primers specific for the human IL-2 sequence (Applied Biosystems, Foster City, CA). The PCR reaction was performed using 37 cycles of denaturation at 94°C for 1 minute, annealing at 60°C for 1 minute, and elongation at 72°C for 1 minute as recommended by Applied Biosystems. The amplified DNA was separated by electrophoresis on 2.5% agarose gels in Tris/Borate/EDTA (TBE) buffer. Jurkat cells were used as a positive control for IL-2 production (52). Amplification of glyceralaldehyde 3-phosphate dehydrogenase (GAPDH) was used to internally normalize the reactions.

## RESULTS

### IL-2 signaling pathways are functional in ALCL cells

**Figure 1:**
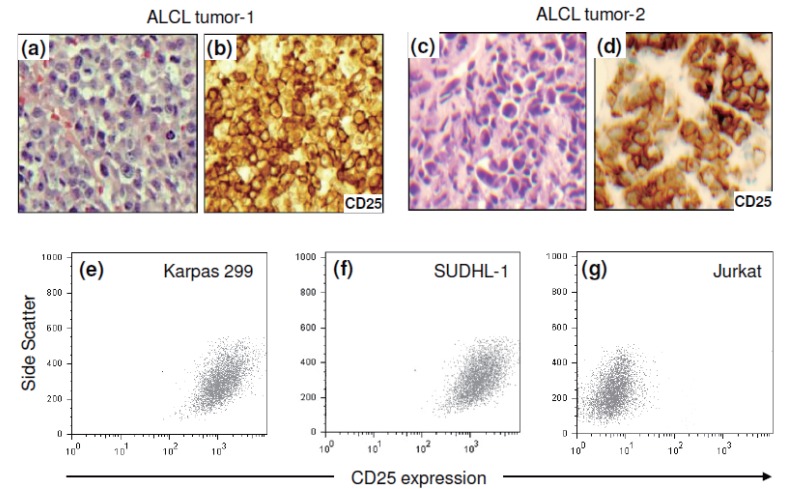
ALCL tumor cells express a high level of CD25, a subunit of high-affinity IL-2 receptors. The formalin-fixed and paraffin-embedded lymphoma tumor tissues from two individual patients, ALCL tumor-1 and ALCL tumor-2, were evaluated for CD25 expression by immunohistochemical studies. (a) and (c), H&E stain of ALCL tumor tissues. (b) and (d) High-level CD25 expression detected in ALCL tumor cells. (e-g) Flow cytometric analysis detected high levels of expression of surface CD25 in ALCL cell lines (Karpas 299 and SUDHL-1 cells) but not in Jurkat cells, a human T leukemia/lymphoma cell line.

To demonstrate IL-2 receptor expression in lymphoma cells, paraffin-embedded tissue sections of tumors from ALCL patients were retrieved from archival files. Immunohistochemical staining for CD25, a subunit of the high-affinity IL-2 receptor, showed high expression levels of IL-2 receptors in lymphoma cells of ALCL tumors (Figure 1a-d). In addition, expression of CD25 in the cultured ALCL cell lines was also examined by flow cytometric analysis. Karpas 299 and SUDHL-1 cells expressed a high level of CD25 (Figure 1e and f). In contrast, little to no CD25 expression was observed in Jurkat cells, a human T-cell leukemia/lymphoma line used as a negative control (Figure 1g).

**Figure 2:**
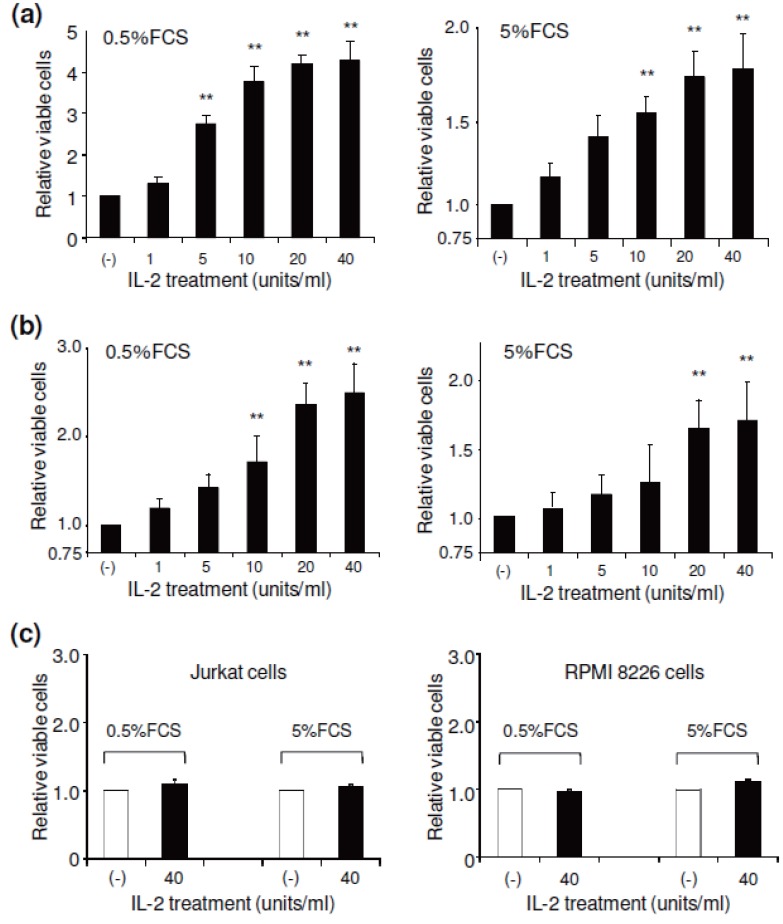
The presence of exogenous IL-2 enhanced ALCL cell growth. To reduce potential effects of serum-derived factor(s), the cells were cultured in medium containing 0.5 and 5% FCS. The cultured ALCL cells, Karpas 299 (a) and SUDHL-1 cell lines (b), were treated with exogenous IL-2 at different concentration as indicated. After culture for 5 days, the relative cell growth rates were calculated by comparing viable cell numbers in each condition and using untreated cultures as a baseline control. As a control, the known CD25-negative Jurkat and RPMI 8226 cell lines (c) were treated under the same conditions and the relative cell growth rates were examined as described above. The experiments were repeated three times and results analyzed by Student’s t-test: **p<0.01 versus controls.

To determine whether IL-2 receptor expression plays a functional role in ALCL cells, we initially examined the role of IL-2 on cell growth. To minimize any potential effects of serum-derived growth factor(s), the cells were cultured in medium supplemented with low concentrations of FCS (0.5 and 5.0%) in the presence of varying concentrations of exogenous IL-2. Five days post addition, cells were harvested, and cell growth rate was evaluated. The presence of exogenous IL-2 significantly stimulated growth of ALCL cells. Indeed, more than a 3-fold increase was observed for Karpas 299 cells and more than a 1.5-fold increase was observed for SUDHL-1 cells under culture conditions containing 0.5% FCS and 40 units/ml of IL-2 (Figure 2a and b), although the effect was less dramatic when cells were cultured with 5% FCS. As a control, Jurkat and RPMI 8226 cells, which are known to be CD25 negative, were cultured and treated under the same conditions. The presence of 40 units/ml of IL-2 in cultures had no effect on the growth of these cells (Figure 2c).

### IL-2 augments the activation of ERK1/2 and enhances the survival of ALCL cells

**Figure 3:**
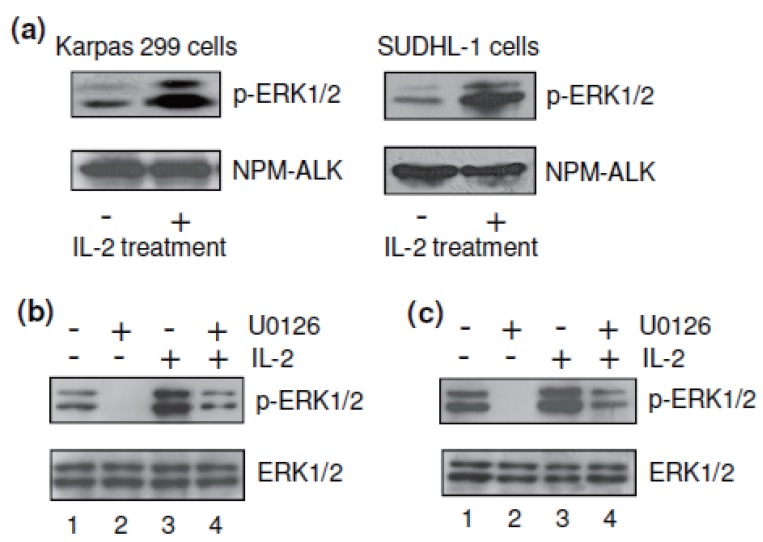
IL-2 augmented phosphorylation/activation of cellular ERK1/2. (a) Western blotting showed that the presence of IL-2 (40 units/ml) in cultures significantly augmented the activation of cellular p-ERK1/2 in Karpas 299 and SUDHL-1 cells but exerted no effect on the protein expression of NPM-ALK as an indicator of equal protein loading. (b) Exposure of Karpas 299 cells to the kinase inhibitor U0126 (5 μM) resulted in complete inhibition of the constitutive ERK1/2 phosphorylation (lanes 1 and 2 of upper panel). IL-2 treatment overcame this U0126-induced decrease of ERK1/2 phosphorylation (lanes 2 and 4 of upper panel). Total ERK1/2 protein levels showed no change in the presence of U0126 and/or IL-2 treatments (lower panel). (c) The similar results were also observed with SUDHL-1 cells under the same treatments. The results are representative of three separate experiments with similar findings.

To investigate the molecular mechanism(s) involved in IL-2 signaling, cultured Karpas 299 and SUDHL-1 cells were treated with 40 units/ml of IL-2 for 30 minutes, and the activation of cellular ERK1/2 proteins was analyzed by western blot using antibody specific for the phosphorylated, active forms (p-ERK1/2). The ERK1/2 signaling pathway showed constitutive baseline activity (Figure 3a), which has been demonstrated to play a critical role in regulating ALCL cell growth (53). Notably, IL-2 treatment augmented phosphorylation and thus the activation of cellular ERK1/2, but had little to no effect on ALK protein expression, which served as an internal loading control. To further determine the precise function of IL-2-mediated ERK1/2 activation in ALCL cells, we used the kinase inhibitor U0126, which is specific for MEK1/2, an upstream kinase in the ERK1/2 kinase cascade (51, 54, 55). Western blot analysis indicated that exposure of Karpas 299 cells to U0126 (5 μM) abrogated the constitutive phosphorylation of ERK1/2 (Figure 3b). The addition of IL-2 to cell cultures increased the level of active ERK1/2 and also overcame the U0126-induced inhibition of ERK1/2 activity in Karpas 299 cells. Similar effects of IL-2 on ERK1/2 phosphorylation were observed in SUDHL-1 cells in the absence of any change in total protein expression of ERK1/2 (Figure 3c).

**Figure 4:**
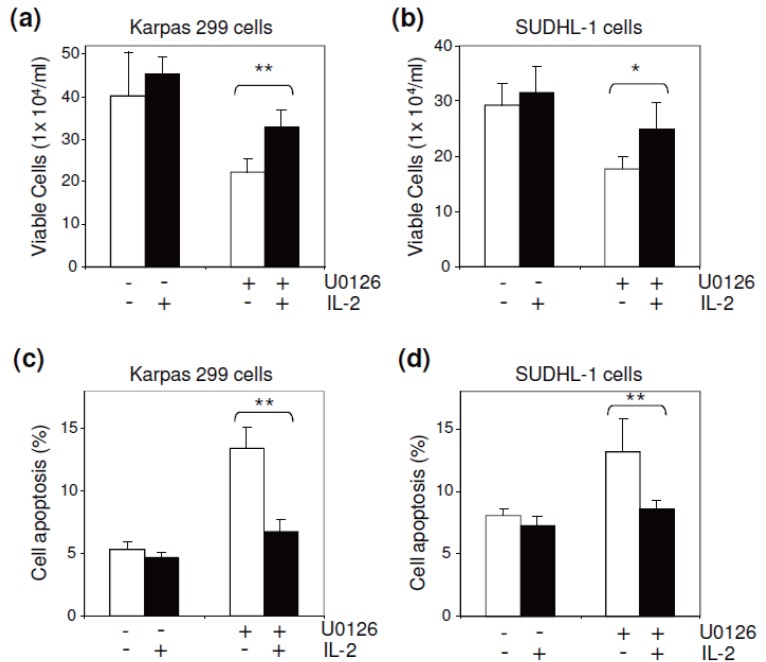
IL-2 regulated ALCL cell growth and apoptosis. To downregulate the ERK1/2 pathway, cultured Karpas 299 (a) and SUDHL-1 cells (b) were exposed to U0126 (5 μM) in the presence or absence of IL-2 (40 units/ml) as indicated. After culture for 36 hours, the number of viable cells in each condition was counted. The presence of exogenous IL-2 significantly reversed the U0126-induced growth arrest of ALCL cells. The treated cells were also stained with FITC-conjugated annexin V, and the apoptotic rate (%) was determined by flow cytometry. The presence of exogenous IL-2 almost completely suppressed the U0126-induced apoptosis of Karpas 299 (c) and SUDHL-1 cells (d). *p<0.05 and **p<0.01 versus controls.

To examine the cellular function of ERK1/2-mediated IL-2 signaling, cultured ALCL cells were exposed to U0126 in the presence or absence of IL-2 for 36 hours. The treated cells were harvested, and viable cells were counted as described above. Inactivation of ERK1/2 by exposure to U0126 resulted in a decreased growth rate in Karpas 299 and SUDHL-1 cells (Figure 4a and b). However, the U0126-induced growth arrest was overcome by the presence of IL-2. In addition, the cell apoptotic rate under each condition was also evaluated with FITC-conjugated annexin V staining and simultaneous flow cytometric analysis. Inactivation of ERK1/2 by U0126 significantly stimulated apoptosis of ALCL cells; however, the presence of IL-2 in cultures largely eliminated U0126-induced cell apoptosis (Figure 4c and d), indicating that ERK1/2-mediated IL-2 signaling plays a role in ALCL cell survival.

### IL-2 is expressed in the tumor microenvironment, but not in ALCL cells

**Figure 5:**
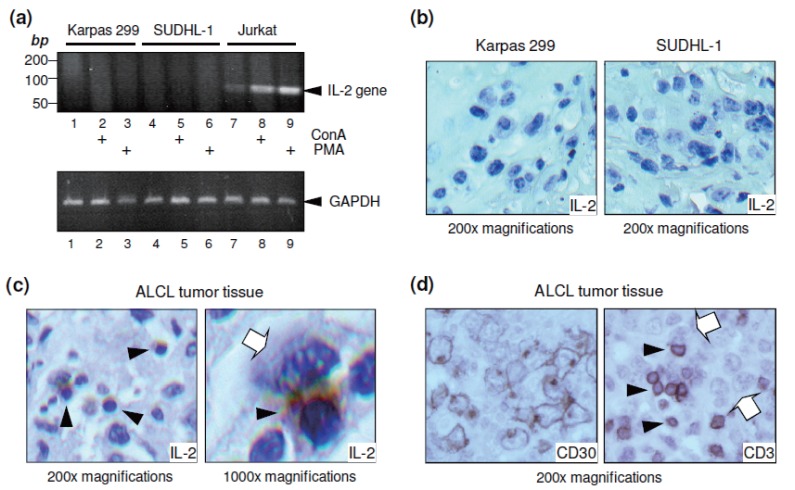
IL-2 protein expressed in background cells of lymphoma tumors, but not in the ALCL cells. (a) Cultured Karpas 299 and SUDHL-1 cells were stimulated with 50 ng/ml of ConA or 10 nM of PMA for 3 hours. As a positive control, Jurkat cells, a human T-cell leukemia/lymphoma cell line known to produce IL-2, were utilized. Cellular IL-2 gene expression was examined by RT-PCR and gel electrophoresis. An 89-bp DNA fragment of the IL-2 gene sequence was detected in Jurkat cells, and this amount was markedly increased by ConA or PMA stimulation (lanes 7-9). In contrast, no IL-2 gene expression was detected in the cultured Karpas 299 and SUDHL-1 cells with or without stimuli (lanes 1-6). As an internal control of equal RNA loading, amplification of GAPDH gene is shown in the lower panel. The results are representative of three separate experiments with similar findings. (b) Immunostaining for IL-2 protein in the cultured ALCL cells was performed on cell block sections as described in “Materials and Methods.” Neither Karpas 299 nor SUDHL-1 cells produced IL-2 protein (100× magnifications). (c) To investigate the potential tissue source of IL-2 within tumor sites, immunostaining for IL-2 protein was performed on formalin-fixed and paraffin-embedded ALCL tumor tissue. IL-2 protein expression was detected in small background cells (solid arrowheads) of lymphoma tissues but not in the large ALCL cells themselves (open arrows) viewed at 100× (left) and 1000× (right) magnifications. (d) To detect any T cells present, the ALCL tumor tissue was stained for CD30 and CD3 separately. The admixed T cells were strongly positive for CD3 (solid arrowheads). In contrast, ALCL tumor cells were not stained for CD3 (open arrows), but were strongly positive for CD30.

Since IL-2 affects ALCL cells, we investigated the potential source of IL-2 for the lymphoma cells. First, cultured Karpas 299 and SUDHL-1 cells were stimulated with ConA (50 ng/ml) or PMA (10 nM) for 3 hours, and IL-2 gene expression was examined. Jurkat cells, a human T-cell leukemia/lymphoma cell line known to produce IL-2, were used as a positive control (52). IL-2 mRNA was then amplified by reverse transcription polymerase chain reaction (RT-PCR) using specific primers. IL-2 gene expression (observed as an 89-bp band) was detected in the Jurkat cells, and the expression was further enhanced by treatment with ConA or PMA (Figure 5a). In contrast, no IL-2 gene expression was detected in stimulated or unstimulated Karpas 299 or SUDHL-1 cells. For further validation, IL-2 protein expression was also examined by immunostaining the fixed ALCL cells. Cultured Karpas 299 and SUDHL-1 cells were stimulated with 10 nM of PMA overnight, and cell blocks were prepared in plasma-thrombin solution, fixed in formalin, and embedded in paraffin. Immunostaining of cell block sections confirmed that Karpas 299 and SUDHL-1 cells themselves did not produce IL-2 (Figure 5b).

The lack of IL-2 gene expression in cultured ALCL cells led us to search for an alternative source of IL-2 within the tumor site. To this end, we performed immunohistochemical studies on formalin-fixed, paraffin-embedded tissue sections from ALCL tumors. Interestingly, immunostaining revealed IL-2 protein expression in the background cells of lymphoma tumors (Figure 5c), but not in the ALCL tumor cells themselves. More importantly, high magnification views showed that the IL-2-expressing cells were often found adjacent to ALCL cells within the lymphoma tumor tissues. These findings indicate that the ALCL cells themselves do not produce IL-2, but rather other cells within the tumor microenvironment may serve as the source of IL-2 in ALCL.

## DISCUSSION

Our study provides the first evidence that IL-2 signaling has physiological consequences in ALCL cells, which express high levels of the high-affinity IL-2 receptor subunit CD25. In addition, molecular and immunochemical studies revealed that IL-2 protein was expressed in the background cells of lymphoma tissues, but not in the ALCL cells themselves. These data suggest that the background cells are a potential *in vivo* tissue source for IL-2 in ALCL tumors. Although the underlying cell type of these background cells is unknown, we observed that these cells are small in size and have condensed chromatin, a slightly irregular nuclear contour, and scant to intermediate cytoplasm. These characteristics are morphologically most compatible with reactive T lymphocytes. The presence of admixed reactive T-lymphocytes in the background is a common feature of ALCL tumors. In contrast to ALCL tumor cells, reactive background T cells were negative for CD30, but strongly positive for CD3 (Figure 5d).

The ERK1/2 pathway is one of major players involved in IL-2 signaling for the regulation of cell functions under physiologic conditions (28-32). In this study, we demonstrate that the addition of exogenous IL-2 increased the activation of ERK1/2 in ALCL cells (Figure 3). Interestingly, our previous studies revealed that silencing of the ALK gene resulted in a partial inhibition of cellular ERK1/2 activity, indicating the presence of an unknown ALK-independent signaling pathway (53). Thus, in ALCL cells, both ALK-dependent and ALK-independent pathways may work in conjunction to regulate ERK1/2 activation and control cellular functions. Moreover, since IL-2 is able to stimulate cells and augment the ERK1/2 kinase pathway, the presence of exogenous IL-2, not surprisingly, enhanced ALCL survival by overcoming the inhibitory effect of U0126, a specific inhibitor to the upstream kinase of ERK1/2 MEK1/2 (51, 54, 55). The detailed mechanism of how IL-2 reversed the effects of U0126 on ERK1/2 activation and whether this action is directly through MEK1/2 are not yet known, but will be the focus of future studies. Notably, we have observed that IL-2 had less of an effect on ERK1/2 activation when cells were treated with 10 μM U0126, and no effect when the final concentration of U0126 was higher than 10 μM. These results may be due to: i) potential non-specific cellular effects resulting from high concentrations of U0126; and/or ii) in addition to IL-2, there may be multiple signaling pathways that contribute to the activation of ERK1/2 in ALCL cells.

Our experiments indicate that the effect of IL-2-induced cell growth was more significant in cultures with lower FCS concentrations (0.5%), and these effects became masked to some extent as serum concentrations increased to 5%. These results may be due to the presence of soluble IL-2 and/or an IL-2-like, serum-derived factor in the FCS, although the levels of such a factor may be sub-functional for normal cells under physiological conditions. ALCL cells, on the other hand, express high levels of CD25, a key component of the high-affinity IL-2 receptors that contribute to the very rapid binding of IL-2 (23, 27). It is important to emphasize that the density of high-affinity IL-2 receptors on the cell surface correlates with IL-2-mediated functional changes. Therefore, since ALCL cells express unusually high levels of high-affinity IL-2 receptors, these cells may be highly sensitive to IL-2 and thus may be able to react to sub-functional concentrations of IL-2 in the serum and/or produced by reactive T lymphocytes within the lymphoma tumor site, resulting in enhanced ALCL cell growth.

Characterization of the IL-2-expressing cells within ALCL tumor sites is critical as these cells serve as an *in vivo* tissue source for IL-2 and may play a pathogenic role in lymphoma development. The analysis of the interactions between lymphoma cells and the microenvironment could provide a basis for the development of alternative treatments aimed at interfering with the survival and/or proliferation signals derived from microenvironment. However, since IL-2 is a cytoplasmic protein and cannot be used as a surface marker for cell sorting, these cells are technically difficult to isolate from lymphoma tissues. In addition, determination of whether ALCL patients have increased serum concentration of IL-2 or IL-2-like factors is also important. To achieve these goals, statistical analysis of a large number of patients with adequate controls is needed. Moreover, further studies are urgently needed to examine the effects of eliminating the *in vivo* source of IL-2 and/or blocking IL-2 signaling in ALCL cells as a potential therapeutic approach to enhance current treatment for ALCL.
